# The Lucifer Match Disease, or Rotting of the Jaw Bone

**Published:** 1859-08

**Authors:** 


### THE LUCIFER MATCH DISEASE, OR ROTTING OF THE JAW BONE.

One of those rare and singular, but hitherto singularly incurable cases of destruction of the lower maxillary bone, by means of fumes of phosphorus, is now in St. Bartholomew's Hospital, where, a few days ago, it was found necessary, as a last resource, to take away the entire jaw-bone completely dead. We have seen so many cases of this fell disorder in the hospitals of London, from time to time, nearly always admitted when they are beyond every means of cure, that we refer to this case rather with an indistinct hope that some ingenuity may be set at work out of doors, amongst our higher class of chemical and medical philosophers, such as those who gave us the safety lamp and vaccination, chloroform, and hints on arsenic in wall paperings, so that these hideous diseases of the lucifer-match manufactories may be prevented, or in default thereof, that the lucifer-match manufactories themselves might be abolished.

Mr. Lawrence, in commenting on this sad case to his class, said that he knew little or nothing of the real nature of the disease, but of the fact of the destruction of this bone, and this bone alone, by the fumes of phosphorus, there is no kind of doubt, as several cases, and all exactly alike, have come under the notice of him and the other surgeons of the hospital. Some of our other London surgeons are inclined to believe that the experiments recently made by M. Ollier on the growth of bone artificially, by the transplantation of the periosteum, may lead, or have led, to some further insight into the nature of this disease. Mr. Paget, we need scarcely say, had come to a similar conclusion from a study of the process of necrosis, and the functions of the periosteum in such cases.

Mr. Quekett has shown that by a special arrangement of rice paper or other thin tissues with lime water, he can imitate the function of what are physiologically termed "raphides," hitherto thought to be as impossible as imitating Haversian canals, cartilage cells, or cancelli; it seems, too, from the experiments of M. Ollier, that a flap of periosteum retaining its attachment by one of its extremities (the rest being lodged in the flesh or muscles) continues to give off bone, much as the rice paper acts, almost like certain vegetable membranes (a piece of onion, for instance,) in giving off raphides and oxalic acid crystals; nay, a strip of periosteum, it is now discovered, may be obtained from the tibia, long enough, according to M. Brown-Séquard, to wind round the bone, or to twist into a perfect corkscrew of bony matter through the muscles—even a new piece of such bone, which with the periosteum has been borrowed, will adhere almost like an epiphysis to the old bone.

So delicate are these processes and operations, that they must be "touched with a gentle hand, for there is a spirit" in the process of true generation that will not bear rough handling; they may be repeated with the periosteum of the same animal on almost every part of his frame, as the deposit of phosphate of lime in the cellular tissue of bone or of muscle goes on alike without any discrimination, so long as the same blood supplies the phosphorus. The periosteum of one animal, however, transplanted to the bones or muscles of another of the same or a different species, invariably dies, or loses its power of keeping up its own periosteal functions! We now come to the lucifer matches. On questioning a poor man, not long ago, at one of our hospitals, he told us that he was employed at Mile End, where all the chief factories of England are situated; his occupation all day was to sit over a pot of melting glue and other ingredients, and to cut sticks of phosphorus into bits the size of a pea, throwing them one by one into the glue. Surgeons all agree that it is through the contact of the phosphorus acid fumes, thus given off, reaching the periosteum of the jaw bones, where it is denuded by loose or absent teeth, that its normal condition of nourishing the bone is altered and finally destroyed. The disease begins in a set of miserable gum-boils, which are lanced, and the bone, with its periosteum, laid bare; the teeth all become loose and fall out, the demon of bad surgery keeping pace with the disease. The case operated on by Mr. Lawrence was of two years' duration, and on admission the poor man presented all the usual familiar marks of this horrible disease. the ordinary fistulous openings leading down to a rotting and diseased periosteum, and a jaw-bone as dead and dry as one might meet in a churchyard, for it is not at all like caries or necrosis.

In something like a dozen of these cases, of which we have now learned the details, the disease commenced quite insidiously, but in the manner just described—gum-boils, and the glue-pot, and loose teeth at first—yet such is the infatuation of these poor men at these works, that they still, at high wages, are content, with these gum-boils and loose teeth, to go on at the employment; too many of the poor fellows, in fact, were inclined to make light of such things. The process of nutrition of the bone, meanwhile, diminishes week by week; there is little or no pain, till the disease finally passes the point of recovery, the whole bone is literally stone dead, and acts as a foreign body would act; the jaw now swells to an enormous size; the health still suffers; cod-liver oil, tonics, &c., are prescribed, but the function of the periosteum, of depositing a special form of phosphate of lime, (according to Professor Graham, composed of two tribasic phosphates, one of these an *alkaline* phosphate,) has been destroyed by the new phosphorous acid fumes or combinations of the lucifer matches.

Mr. Lawrence, in referring to the perfectness of the process by which the regeneration of the living bone is usually brought about in cases of common necrosis—as remarked, too, by Mr. Paget, still, if possible, more complete in the lower invertebrate animals than in man himself,—could not help wondering at the total and incurable destruction of the jaw-bone that is worked by the acids of the lucifer-match process. In one case, indeed, we observed the surgeon, on a "taking-in" day, seeing the poor man for the first time and examining the swollen jaw, to pick out the entire bone with his fingers, to the entire dismay of the poor patient. It was as dead as a fossil, but as perfect; this has been seen more than once in the hospitals.

At Nuremberg, in Germany, we were told by Mr. Paget, the great centre in Continental Europe for the formation of "lucifers," this horrible disease is also very common, and has been very fatal; it is a disease not in any shape or at all likely to be seen in small towns; nor do we see why some legislative precaution should not be enforced, to banish it altogether from our hospitals. Of one thing there can be no doubt: that the poor men employed in such factories do not at all understand the danger they are exposed to, and some obvious prophylactic measures are systematically neglected. —*Medical Circular*, *May* 18.

